# Respiratory function of children and adolescents with osteogenesis imperfecta: respiratory muscle strength, forced vital capacity, and peak expiratory flow

**DOI:** 10.1590/1984-0462/2023/41/2022092

**Published:** 2023-03-13

**Authors:** Patricia de Abreu Farias Carvalho, Taiane Sousa Regis, Adriana Virgínia Barros Faiçal, Regina Terse-Ramos, Angelina Xavier Acosta

**Affiliations:** a Universidade Federal da Bahia, Salvador, BA, Brasil.

**Keywords:** Osteogenesis imperfecta, Respiratory function tests, Lung diseases, Osteogênese imperfeita, Testes de função respiratória, Doença pulmonar

## Abstract

**Objective:**

This study aims to evaluate the respiratory function of children and adolescents with osteogenesis imperfecta (OI) followed up at a referral center.

**Methods:**

A cross-sectional study was conducted with a non-probabilistic sample. Manovacuometry was performed with the measurement of maximal inspiratory pressure (MIP) and maximal expiratory pressure (MEP), and in addition, peak expiratory flow (PEF) and ventilometry were performed to measure forced vital capacity (FVC).

**Results:**

In total, 23 individuals were evaluated, with a mean age of 11.6±3.4 years, 56.5% of whom were females. Regarding the classification of OI, 56.5% of the sample belonged to type IV, 30.5% to type III, and 13% to type I. The mean MIP was 64.4% of the predicted, and the mean MEP was 56.2% of the predicted. Overall, the mean PEF was 213.9 L/min, but only 140.6 L/min in the OI type III group. Median FVC was 1.9 L, corresponding to 110% of the predicted.

**Conclusions:**

Respiratory function of the study subjects was altered, with respiratory muscle strength values lower than expected in the whole sample, and peak expiratory flow was significantly reduced in the OI type III group.

## RESUMO

**Objetivo:**

Avaliar a função respiratória de crianças e adolescentes com osteogênese imperfeita (OI) acompanhados em um centro de referência.

**Métodos:**

Realizou-se um estudo de corte transversal, com amostragem não probabilística. Foi realizada manovacuometria com mensuração da pressão inspiratória máxima (PIM) e pressão expiratória máxima (PEM), além do pico de fluxo expiratório (PFE) e da ventilometria para a medida da capacidade vital forçada (CVF).

**Resultados:**

Foram avaliados 23 indivíduos, com média de idade de 11,6±3,4 anos, sendo 56,5% do sexo feminino. Com relação à classificação da OI, 56,5% da amostra pertencia ao tipo IV, 30,5% ao tipo III e 13% ao tipo I. A média de PIM foi de 64,4% do previsto, e a PEM foi de 56,2% do previsto. A média de PFE foi de 213,9 L/min, sendo 140,6 L/min no grupo de OI tipo III. A mediana da CVF foi de 1,9 L, correspondendo a 110% do previsto.

**Conclusões:**

A função respiratória dos indivíduos estudados encontrava-se alterada, com valores abaixo do esperado em toda a amostra para força muscular respiratória, além do PFE reduzido no grupo OI tipo III.

## INTRODUCTION

Osteogenesis imperfecta (OI) is a rare genetic disease characterized by irregularities in the amount, structure, or processing of type 1 collagen. The estimated prevalence is 1 in 15,000 to 20,000 births.^1,2^ Variants with dominant effect alter the *COL1A1* or *COL1A2* genes.^3^ Sillence et al. classified OI into four types based on clinical data and genetic findings, with severity ranging from mild to fatal.^4^ Clinical manifestations include osteopenia, more or less pronounced short stature, skeletal deformities, dentinogenesis imperfecta, joint laxity, and blue sclera.^5^

Limitation of respiratory function is a secondary manifestation of the biomechanical changes in the thorax present in OI and the main cause of morbidity and mortality, especially in the most severe forms of the disease. Skeletal abnormalities affecting the chest wall result in diaphragmatic constraint, which decreases alveolar ventilation due to pulmonary compression.^6,7,8^ In addition, intrinsic impairment of the lung has been studied in more detail because type I collagen is essential for the development of the lung parenchyma and, consequently, defects in type I collagen impair lung function.^9^

Few studies have examined respiratory function in children and adolescents, using only spirometry as an instrument. The literature lacks studies on the use of respiratory assessment instruments, such as ventilometer, manovacuometer, and peak flow, which are readily available in hospitals and outpatient settings. Such studies could guide physical respiratory therapy for these individuals by providing data on respiratory muscle strength, lung volume, and lung capacity. These parameters are often impaired in the most severe forms of OI and are important measures of treatment success. The purpose of this study was to evaluate respiratory function with ventilometer, manovacuometer, and peak flow in children and adolescents with OI in a referral center.

## METHOD

An observational, descriptive, and cross-sectional study was conducted at an OI referral center from October 2019 to October 2021. A non-probabilistic sample of 24 participants with a confirmed OI diagnosis, aged 6–21 years, was recruited. One participant who did not understand the instructions of the assessment instruments was excluded, making data collection infeasible. Thus, 23 individuals participated in the study. This number represents 40% of the eligible population at this referral center. Data collection was performed by two researchers trained in the assessment tools used and took approximately 30 min during the hospitalization for treatment with parenteral bisphosphonates at the Pediatrics Clinic. Sociodemographic data were collected using a questionnaire developed by the authors and by reviewing medical records.

A respiratory assessment was performed with the participant seated. Maximal inspiratory pressure (MIP) and maximal expiratory pressure (MEP) were measured using a manovacuometer (Ventbras Ind. Bras.), with occlusion of the nose by a nose clip. MIP was measured with a maximal inspiratory effort, starting from the residual volume, and MEP was measured with a maximal expiratory effort, starting from the total lung capacity. The highest value among the three attempts was considered. For the analysis of respiratory muscle strength, the reference equation for respiratory pressures in the Brazilian pediatric population proposed by Lanza et al. was used. For this calculation, the factors considered are sex, age, weight, and height.^10^

Peak expiratory flow (PEF) was measured using the Peak Flow Meter (Medicate^®^), with a forced expiration starting from inspiration at the level of total lung capacity. To assess forced vital capacity (FVC), a ventilometer (nSpire™ Wright^®^ Mk8) was used. The participants were instructed to take a deep breath to their maximum capacity, hold the air for 1–2 s, and then exhale with maximum effort. The highest value among the three attempts was considered. For the analysis of FVC, the reference values for spirometry in Brazilian children, proposed by Jones et al., were used. For the calculation, the factors considered are sex, age, height, and skin color.^11^

Categorical variables were presented in absolute and relative frequencies. The measures of central tendency and dispersion were presented as mean and standard deviation or median and interquartile range. To assess normality, the Kolmogorov-Smirnov test was used. Data analysis was performed using the Statistical Package for the Social Sciences (SPSS) software, version 21.

The project was approved by Ethics Committee (decision number 3.020.967). All patients and/or guardians signed the free and informed consent form, in addition to the assent form, when indicated.

## RESULTS

In total, 23 individuals with OI were evaluated, of whom 56.5% were female. The mean age (standard deviation) was 11.6 (3.4) years. Most individuals (95.7%) were from countryside cities. Regarding the classification of OI, 13% were type I, 30.5% were type III, and 56.5% were type IV. The sociodemographic and clinical characteristics of the participants are shown in Table 1. Musculoskeletal alterations in the thorax and spine were observed, being more pronounced in the OI type III. It was impossible to obtain a history of rib fractures at birth or during life because of inaccurate or incomplete records and memory bias.

**Table 1.** Sociodemographic and clinical characteristics of children and adolescents with osteogenesis imperfecta evaluated in this study (Bahia, Brazil, October 2019 to October 2021).

Variables	Values
Age (years)^*^	11.6 (3.4)
Gender^†^	
Male	10 (43.5)
Female	13 (56.5)
Residency^†^	
Countryside cities	22 (95.7)
Capital	1 (4.3)
Family income^†^	
Up to 1 minimum wage	19 (82.6)
1–3 minimum wage	4 (17.4)
Z score		
Weight for age^*^	-2,08 (2.25)
Height for age^*^	-3.7 (0.5)
BMI for age^‡^	1.09 (-0.29–1.94)
OI classification†	
I	3 (13)
III	7 (30.5)
IV	13 (56.5)
Need of walking devices†	
Totally dependent	2 (8.7)
Wheelchair-bound	10 (43.5)
Need of walker or crutches	5 (21.7)
Walking independently	6 (26.1)
Regular physiotherapy^†^	
Yes	2 (8.7)
No	21 (91.3)
Duration of treatment at OIRC (years)^‡^	4.2 (2.9–5.7)

BMI: body mass index; OI: osteogenesis imperfecta; OIRC: osteogenesis imperfecta referral center. ^*^Values presented as mean and standard deviation; ^†^Values presented as n (%); ^‡^Values presented as median and interquartile range.

The overall mean respiratory muscle strength score was lower than the predicted percentage, with a lower score for expiratory strength. The values for respiratory muscle strength, FVC, and PEF are shown in Table 2.

**Table 2.** Assessment of respiratory muscle strength, PEF, and FVC in children and adolescents with osteogenesis imperfecta evaluated in this study (Bahia, Brazil, October 2019 to October 2021).

Variables	Overall mean/median	% predicted	Type I (n=3)	Type III (n=7)	Type IV (n=13)
			% Predicted		
MIP^*^ (cmH_2_O)	67.6 (27.3)	64.4 (22.4)	73.0 (15.8)	58.6 (24.5)	65.5 (23.2)
MEP^*^ (cmH_2_O)	57.3 (18.3)	56.2 (15.9)	64.9 (29.5)	53.7 (11.4)	55.5 (15.2)
FVC (L)^†^	1.9 (0.7–2.7)	110 (89.7–129.2)	120 (116–129)	100 (85–105)	114 (87–156)
			Absolute values		
PEF (L/min)^*^	213.9 (87.7)	-	206.7 (65.1)	140.6 (48.3)	255.0 (85.2)

MIP: maximum inspiratory pressure; MEP: maximum expiratory pressure; FVC: forced vital capacity; PEF: peak expiratory flow. *Values are presented as mean and standard deviation; †Values presented as median and interquartile range.

The presence of other pulmonary diseases was interrogated, and only one participant reported asthma. The participants did not report chest pain before, during, or after the respiratory assessment, so it did not affect the assessments.

## DISCUSSION

Given the pathophysiology of OI, which causes significant changes in respiratory function in the most severe forms, assessment of respiratory function is essential to guide clinical and physical therapy interventions. In this study, respiratory function was assessed in children and adolescents with OI, with a high prevalence of respiratory muscle weakness and significant reductions in PEF in the group with type III OI. Although the respiratory disease is considered the leading cause of death in this population, accounting for 81.6% of deaths in type III OI and 39% in types I and IV, there are few studies on this topic.^12^

Muscle strength, both inspiratory and expiratory, was lower than expected in the entire sample studied, being more pronounced in the OI type III, followed by the type IV. Data for comparison are lacking in the literature. The combination of *pectus carinatum,* fragile ribs, and spinal deformity presents a mechanical disadvantage to the muscles of the thorax, which may explain this result.^13^

The overall median FVC in the studied sample was 1.9 L, which is 110% of the predicted value. In a multicenter study, Tam et al. evaluated spirometric data from 217 individuals, including children and adults with OI, and found that both men and women with type III OI had lower FVC and FEV1 than those with OI types I and IV, with larger differences between the second and fourth decades of life.^14^ LoMauro et al. observed adolescents and adults with OI using spirometry and found that FVC and FEV1 were lower than predicted in both types III and IV.^13^ Our results differ from these studies: a restrictive pattern was not present, possibly because the expansion capacity of the rib cage is preserved, generating the negative pressure needed to produce inspiratory flow and thus maintaining the viscoelastic properties of the lung. Tam et al.^14^ evaluated a large sample and thus had a greater opportunity to demonstrate differences between groups. LoMauro et al.^13^ studied only patients with moderate and severe OI, which led to the inclusion of patients with a higher degree of respiratory abnormalities. It is important to emphasize that both studies evaluated only spirometric data.

The absolute value of PEF was lower in the type III OI, with a mean value (SD) of 140.6 (48.3) L/min. The weakness of the expiratory muscles can also cause reduced expiratory flow and cough flow. The PEF analysis was not performed using the predicted percentage because equations are not available for the OI population. Furthermore, the equations used for the general population do not apply to the study sample, since some individuals have extremely short stature. The PEF is the most reproducible method for measuring cough severity and assessing the risk of pulmonary complications in patients with neuromuscular diseases who have a restrictive ventilatory pattern, which also occurs in the most severe forms of OI.^15^

In studies examining patients with neuromuscular diseases, a PEF of less than 160 L/min was ineffective in keeping the airway clear.^16^ Patients who produced a PEF of 270 L/min had a low risk of developing respiratory failure during airway infection.^16,17,18^ The use of readily available clinical practice tools allows for a thorough assessment of the respiratory function in this population and helps to develop a multi-professional care plan and respond early to functional changes. The lack of predictive models that account for factors that affect respiratory outcomes in children and adolescents with OI, such as extremely short stature and chest deformities, is an important gap in the interpretation of results and should stimulate further research in this area.

There are some limitations to this study. It was not possible to evaluate all eligible patients, mainly because of the difficulty of participants in continuing treatment during the SARS-CoV-2 pandemic. Computed tomography of the chest was not performed to assess structural changes and volume reductions in the lungs, such as atelectasis. Other tests to assess lung volume were not performed, such as spirometry, maximal nasal inspiratory pressure, and peak cough flow. There were no records of arterial blood gas analysis of the participants.

Respiratory function was altered in the children and adolescents with OI studied, with respiratory muscle strength values lower than predicted for the entire sample. In addition, PEF was reduced in the group with type III OI. Regarding strength, this study demonstrated that simple respiratory assessment tools are easily reproducible and well tolerated by participants. Despite the limitations, given the results of this study, we reiterate that follow-up of individuals with OI requires assessment and treatment of conditions related to the neuromusculoskeletal and respiratory systems. Given the lack of assessment and intervention studies targeting respiratory changes in patients diagnosed with OI, this study highlights the need to develop future research for this purpose.
